# CRISPR-Cas13a-Based Lateral Flow Assay for Detection of Bovine Leukemia Virus

**DOI:** 10.3390/ani14223262

**Published:** 2024-11-13

**Authors:** Yuxi Zhao, Jingwen Dai, Zhen Zhang, Jianguo Chen, Yingyu Chen, Changmin Hu, Xi Chen, Aizhen Guo

**Affiliations:** 1 National Key Laboratory of Agricultural Microbiology, Hubei Hongshan Laboratory, College of Veterinary Medicine, Huazhong Agricultural University, Wuhan 430070, China; yuxiz@webmail.hzau.edu.cn (Y.Z.); chenjg@mail.hzau.edu.cn (J.C.); chenyingyu@mail.hzau.edu.cn (Y.C.); hcm@mail.hzau.edu.cn (C.H.); chenxi@mail.hzau.edu.cn (X.C.); 2 Key Laboratory of Development of Veterinary Diagnostic Products, Key Laboratory of Ruminant Bio-Products, China Ministry of Agriculture and Rural Affairs, Huazhong Agricultural University, Wuhan 430070, China; 3 College of Animal Science and Technology, Henan Agricultural University, Zhengzhou 450046, China; daijingwen0264@163.com; 4 Henan Seed Industry Development Center, Zhengzhou 450045, China

**Keywords:** RAA, CRISPR-Cas13a, lateral flow strip, bovine leukemia virus, detection

## Abstract

Bovine leukemia virus (BLV) is the causative agent of enzootic bovine leukosis (EBL), which presents worldwide prevalence. BLV caused substantial economic loss in China around the 1980s; then, it could not be detected for some time, until recently. Due to its latent and chronic characteristics, the efficient and accurate detection of BLV is of utmost significance to the timely implementation of control measures. Therefore, this study harnessed the recombinase-aided amplification (RAA), clustered regularly interspaced short palindromic repeats (CRISPR)-associated protein 13a (Cas13a) technology, and lateral flow (LF) strips to develop an efficient method for detection of BLV. In this method, isothermal amplification of the targeted *pol* gene is performed at 37 °C with a detection threshold of 1 copy/µL, and the procedure is completed in 100 min. This assay demonstrated high selectivity for BLV, as indicated by the absence of a cross-reaction with six common bovine pathogens. Remarkably, 100 blood samples from dairy cows were tested in parallel with a conventional quantitative polymerase chain reaction (qPCR) and this method and the results showed 100% agreement. Furthermore, this method exhibited good repeatability. In conclusion, in this study, we established a sensitive and specific method for BLV detection, which shows promise for application in BLV surveillance.

## Simple Summary

Cattle farmers worldwide face a serious challenge from a virus called bovine leukemia virus, which harms cow health and farm profits. Currently, the best way to control this virus is to find infected cows and remove them from herds, as there are no effective treatments or vaccines. While there are good tests for finding this virus, they often need expensive lab equipment. Our team created a new test that is accurate and does not need complex machines. This test combines three scientific techniques to detect the virus’s genetic material easily. We found that our method performs well comparably to existing testing methods. This technology shows promise for development as a pen-side testing tool in the future. Such development could provide valuable new options for monitoring bovine leukemia virus, helping farmers better protect herd health and supporting livestock industries worldwide.

## 1. Introduction

Bovine leukemia virus (BLV) is an oncogenic member of the *Deltaretrovirus* genus within the *Retroviridae* family and closely related to human T-lymphotropic virus [1]. BLV is responsible for causing enzootic bovine leukosis (EBL), which is the most common infectious neoplastic disease observed in cattle worldwide, except for most Western European countries that have eradicated EBL [2]. While infected cattle usually become asymptomatic carriers, approximately 30% develop persistent lymphocytosis, and 5% die from malignant lymphoma [3]. Moreover, infection with BLV is associated with a decrease in milk production, a shortened lifespan, and immune suppression [4,5,6]. Recently, several reports have linked BLV with human breast cancer because of an association between higher prevalence of the BLV provirus and the occurrence of this disease [7,8,9]. These findings suggest the potential direct threat posed by BLV infection in cattle to public health.

The efficient detection of BLV in surveillance is an essential measure in the prevention and control of this infectious disease [10], including curbing virus dissemination and mitigating potential harm to the cattle industry. Conventional diagnostic methods for BLV infection include the culture-based method [11], serological testing [12], and molecular diagnosis [13]. Although the culture-based method offers the potential for the additional and extensive testing of BLV, it is inherently limited due to its time-consuming process and low sensitivity [14]. While the serological test is commonly used due to its convenience, it cannot reliably detect early infections [15]. On the other hand, molecular approaches such as a quantitative polymerase chain reaction (qPCR) and nested polymerase chain reaction (nPCR) are powerful diagnostic tools due to their high sensitivity and specificity [16]. BLV–Coordination of Common Motifs–qPCR-2 (BLV-CoCoMo-qPCR-2) targeting the *LTR* gene is widely used for BLV detection [17]. However, the requirement for expensive and sophisticated thermal cyclers poses a limitation in basic laboratory settings.

Consequently, alternative methods based on isothermal amplification technology, such as nuclear acid sequence-based amplification [18], loop-mediated isothermal amplification (LAMP) [19], recombinase polymerase amplification (RPA) [20], and recombinase-aided amplification (RAA) [21], have been developed for pathogen detection without the requirement for thermal cyclers such as a PCR and qPCR. Recently, clustered regularly interspaced short palindromic repeats (CRISPR)-associated proteins (Cas), such as Cas12 (Cas12a and Cas12b) [22,23], Cas13 (Cas13a and Cas13b) [24,25], and Cas14 [26], have been actively used to develop new detection methods based on their collateral cleavage activity after recognizing their respective targets. A recently developed, notable molecular detection platform called SHERLOCK combines the amplification of RPA with the transcription of the amplified DNA into RNA by using the T7 system and the collateral effect of CRISPR-Cas13a [27,28]. Coupled with efficient isothermal assays and visual readouts, the CRISPR-Cas13a-based diagnostic approach allows for rapid on-site detection. This principle has been applied to the detection of various pathogens, including Lassa virus [29], African swine fever virus [30], and porcine circovirus type 4 [31].

The objective of this study was to develop an efficient detection system for BLV that integrates CRISPR-Cas13a, RAA, and lateral flow (LF) strips. To achieve this goal, we meticulously designed primers and probes by targeting the conserved sequences of both the *pol* and *env* genes. As a result, this method presents a viable approach for the detection and timely monitoring of BLV with high sensitivity and specificity.

## 2. Materials and Methods

### 2.1. Plasmids, Bacteria, Viruses, and Clinical Blood Samples

Partial *pol* and *env* gene sequences were first synthesized and then subcloned into the pUC57 plasmid to generate the recombinant constructs pUC-*pol*1 (GeneBank accession numbers: MH170028), pUC-*env* (GeneBank accession numbers: LC733340), and pUC-*pol*2 (GeneBank accession numbers: LC080651), with *pol*1 and *pol*2 representing two different fragments of the *pol* gene. These constructs served as the detection targets unless otherwise specified. The recombinant plasmids were subsequently transformed into *Escherichia coli* (*E. coli*) DH-5α for propagation. The successful construction of these plasmids was confirmed with restriction enzyme digestion analysis and sequencing to ensure the accuracy and integrity of the inserted sequences. The gene synthesis and plasmid construction procedures were performed by Wuhan AuGCT Biotechnology Co., Ltd. (Wuhan, China).

We used a total of 100 EDTA-anticoagulated whole-blood samples obtained for a previous, unpublished study conducted by our research team. To further examine the specificity and cross-reactivity of our assay, we leveraged a panel of six common bovine pathogens that had been previously isolated and preserved in our laboratory. This panel included extracted genomes from both viral and bacterial agents of significant veterinary importance: bovine viral diarrhea virus (BVDV), bovine coronavirus (BCoV), *E. coli*, lumpy skin disease virus (LSDV), *Salmonella*, and *Cryptosporidium*.

### 2.2. RAA Primer Design and crisprRNA (crRNA) Preparation

To identify the specific primers and crRNA, we constructed a local database containing 20 sequences of the three genes retrieved from GenBank. The conserved sequence of each gene was identified by performing multiple alignments with Mafft (version 7). The RAA primers were selected from the conserved nucleotide regions of the three genes based on the RAA primer design requirements. Additionally, a T7 promoter sequence (5′GAAATTAATACGACTCACTATAGGG3′) was appended to the 5′-end of the RAA forward primer [31]. Three primer sets were designed, as shown in Table 1.

To target the RAA products of three genes, crRNAs specific to each gene were designed for Cas13a. To prepare them, DNA templates were appended with a T7 promoter sequence and synthesized as primers by Sangon Biotech (Table 2). Two primers were annealed to create double-stranded DNA by using annealing buffer (Solarbio, Beijing, China), which was then purified with gel extraction. The double-stranded DNA was transcribed to crRNA, following the instructions of the HiScribe T7 Quick High Yield RNA Synthesis Kit (NEB, Ipswich, MA, USA). The resulting crRNA was subsequently purified by using Agencourt RNAClean XP (Beckman Coulter, Brea, CA, USA) as per the manufacturer’s recommended protocol and then stored at −80 °C until use [30].

### 2.3. Evaluation and Optimization of RAA Reactions

RAA reactions were performed by using an RAA kit (obtained from Anhui Microanaly Genetech Co., Ltd., Hefei, China), following the manufacturer’s instructions. Briefly, 50 µL of the reaction mixture contained 25 µL of buffer A, 15.5 µL or 16.5 µL of nuclease-free water (depending on the condition), 1 µL of DNA template, 1 µL of RAA polymerase, 2 µL of *pol*1/*env*/*pol*2-F (forward primer; 10 µM), 2 µL of *pol*1/*env*/*pol*2-R (reverse primer; 10 µM), 2.5 µL of magnesium acetate, and 1 µL of 25× SYBR Green I. The reactions were incubated at 37 °C in a Smart016 instrument (Hefei Yuanzai Biotechnology Co., Ltd., Hefei, China), and fluorescence data were collected 160 times to select the most effective primer pairs [32]. Then, the RAA reaction products were transferred to the CRISPR-Cas13a cleavage assay and were then incubated at 37 °C for 40 min without the addition of 25× SYBR Green I.

### 2.4. CRISPR-Cas13a-LF Reaction

For BLV detection, a CRISPR-Cas13a-LF assay was developed by integrating RAA, LwaCas13a, and an LF. The LF utilized a biotin-tagged FAM-RNA reporter. In the negative control setup, an anti-FAM antibody conjugated to gold nanoparticles bound to the FAM-RNA-biotin reporter, and this conjugate was captured by a biotin ligand at the control line. In the positive control scenario, cleavage of the FAM-RNA-biotin reporter occurred, resulting in the accumulation of the anti-FAM antibody–gold nanoparticle conjugate at the test line and a corresponding reduction at the control line [31].

**Figure 1:**
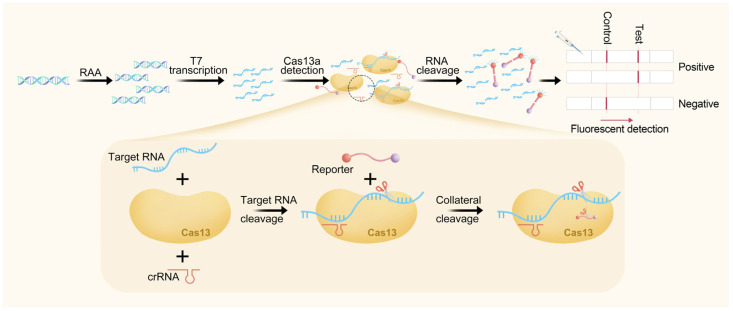
Schematic representation of the CRISPR-Cas13a-LF BLV test workflow. The BLV genome was extracted from bovine blood samples, amplified by RAA, and transcribed into RNA to activate the Cas13a nuclease. The activated nuclease then recognized crRNA and cleaved the reporter molecule, resulting in a visible band on the test strip. The presence of either the test line alone or both the test and control lines indicates a positive result, whereas the presence of only the control line indicates a negative result. RAA = recombinase-aided amplification.

The CRISPR-Cas13a-LF reaction system comprised 50 µL of 45 nM LwaCas13a (Magiltd, Hefei, China), 22.5 nM crRNA, 125 nM RNA reporter, 0.25 µL RNase inhibitor, 1 mM dNTP, 0.4 µL of T7 RNA Polymerase Mix (NEB, Ipswich, MA, USA), and 1 µL of RAA-amplified product, and nuclease-free water was used as a negative control instead of the RAA reaction products. The reactions were performed at a constant temperature of 37 °C for 40 min. Following incubation, a volume of 10 µL of the product was diluted 10-fold with a Hybridetect assay buffer and subsequently 10 µL of the diluted solution was applied to LF strips (obtained from Hefei Yuanzai Biotechnology Co., Ltd., Hefei, China) utilizing a biotin-tagged FAM-RNA reporter for analysis. The results were read and recorded after a brief incubation period of 3–5 min. The appearance of a specific line indicated a positive result [30]. Figure 1 provides a schematic representation of the CRISPR-Cas13a-LF.

### 2.5. Analytical Sensitivity and Specificity of RAA-Cas13a-LF

To test for analytical sensitivity, 10-fold serially diluted plasmid DNA templates ranging from 1 copy/µL to 10^10^ copies/µL were prepared, and detection was performed by using the RAA-Cas13a-LF assay.

To verify the analytical specificity of the proposed assay, the nucleic acids from six bovine common pathogens, including BVDV, BCoV, LSDV, *E. coli*, *Salmonella*, and *Cryptosporidium*, were subjected to detection.

### 2.6. Comparison of RAA-Cas13a-LF and BLV-CoCoMo-qPCR-2 Assays for Detection of Field Blood Samples

Nucleic acids were extracted from 100 bovine whole-blood samples by using an M5 HiPer Blood Genomic DNA Mini Kit with columns, following the manufacturer’s instructions (Mei5bio, Beijing, China). We tested them in parallel by using both RAA-Cas13a-LF and BLV-CoCoMo-qPCR-2 assays [17]. Then, agreement between the two methods was assessed.

## 3. Results

### 3.1. Screening of Optimal RAA Primers

**Figure 2:**
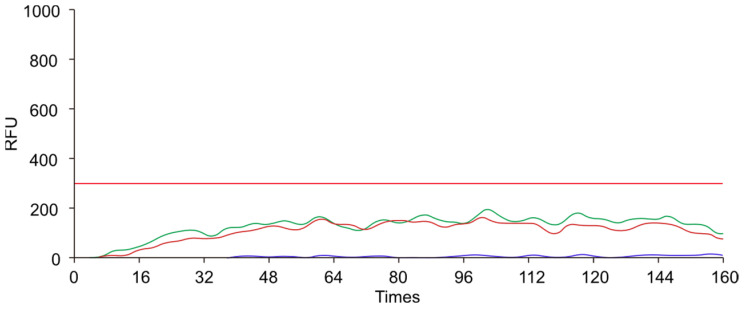
The amplification curves obtained by using the *pol*1 primer. The blue curve represents the negative control, while the green and red curves represent two replicates. The red horizontal line represents the threshold line. The x-axis represents the number of fluorescence collections, and the y-axis represents the fluorescence values. RFU: Relative Fluorescence Units.

**Figure 3:**
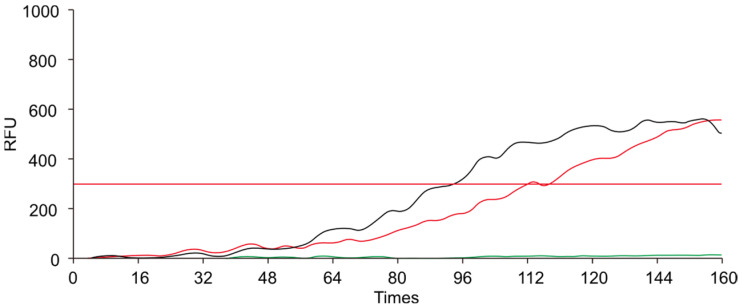
The amplification curves obtained by using the *env* primer. The green curve represents the negative control, while the red and black curves represent two replicates. The red horizontal line represents the threshold line. The x-axis represents the number of fluorescence collections, and the y-axis represents the fluorescence values. RFU: Relative Fluorescence Units.

**Figure 4:**
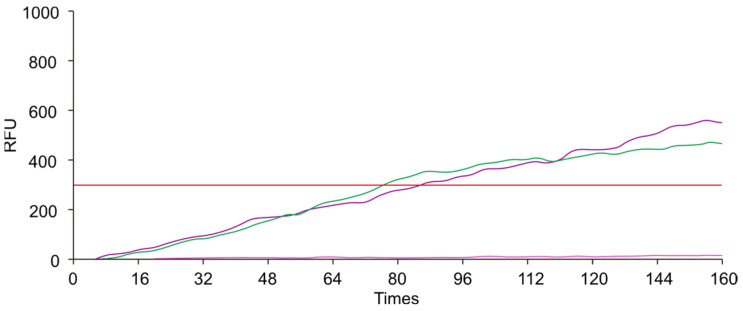
The amplification curves obtained by using the *pol*2 primer. The pink curve represents the negative control, while the green and purple curves represent two replicates. The red horizontal line represents the threshold line. The x-axis represents the number of fluorescence collections, and the y-axis represents the fluorescence values. RFU: Relative Fluorescence Units.

To ensure specificity and efficient amplification, we designed a pair of primers targeting the conserved sequences. We evaluated the performance of different RAA primers by conducting amplification reactions with a qPCR instrument. As illustrated in Figure 2, Figure 3 and Figure 4, the *env* and *pol*2 primers exhibited clear amplification curves, whereas the *pol*1 primer did not. Therefore, the subsequent experiments focused on assessing the performance of the *env* and *pol*2 primers.

### 3.2. Establishment of the CRISPR-Cas13a-LF Assay

**Figure 5:**
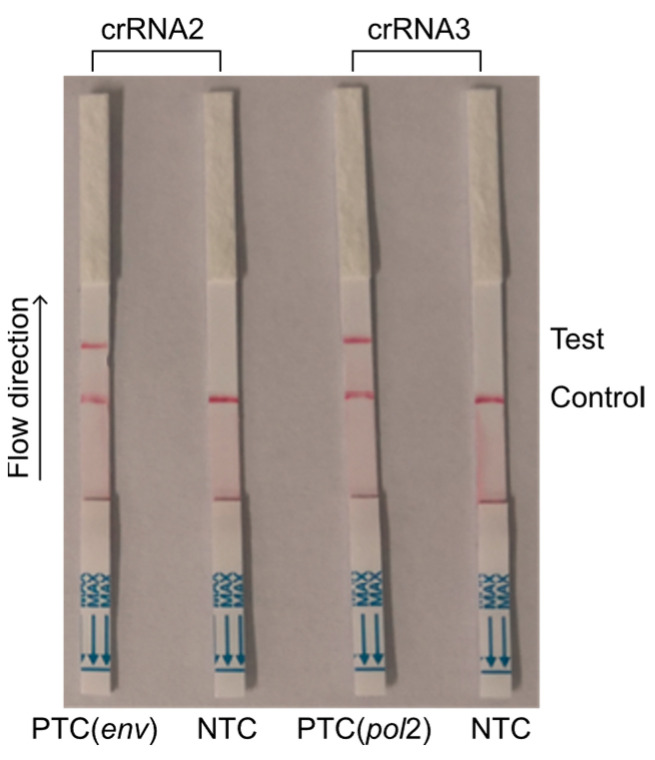
CRISPR-Cas13a-LF detection for validation of crRNAs. PTC (*env*), pUC57 *env* plasmid; PTC (*pol*2), pUC57 *pol*2 plasmid; NTC, negative control.

The practical workflow consisted of three steps. Firstly, we employed RAA to amplify the target gene from bovine blood samples and transcribe it into single-stranded RNA (ssRNA). Secondly, the LwaCas13a nuclease recognized the crRNA sequences designed for each of the two Cas13a crRNAs, which targeted the RAA-amplified products of the target gene, and cleaved the reporter molecule. Finally, the presence of the reporter molecule was visualized as a band on the test strip. Among the two designed Cas13a crRNAs, the combination of crRNA2 and crRNA3 was selected for further performance optimization of the CRISPR-Cas13a-LF assay (Figure 5).

### 3.3. Sensitivity Analysis

**Figure 6:**
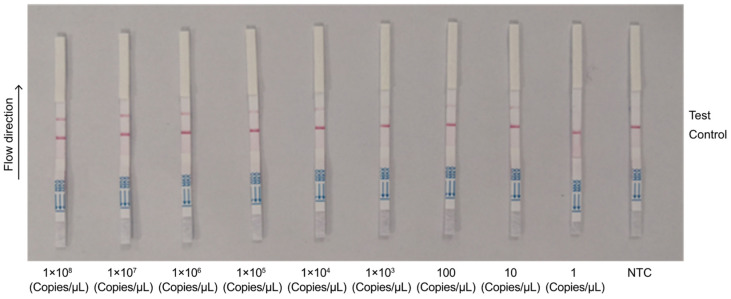
Sensitivity detection of *env* primer combined with crRNA2. Plasmid diluted with a 10-fold gradient: 1 × 10^8^ copies/μL–1 × 1 copy/μL; NTC represents the negative control; Control represents the control line; Test represents the test line.

**Figure 7:**
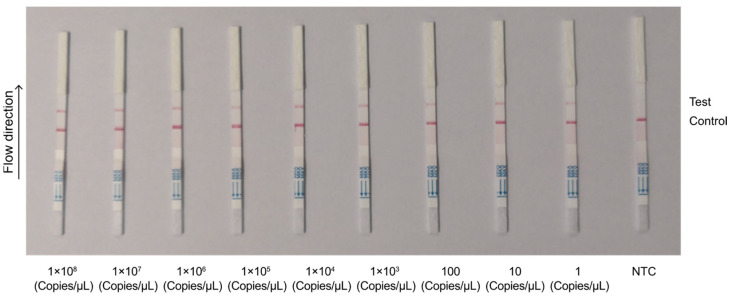
Sensitivity detection of *pol*2 primer combined with crRNA3. Plasmid diluted with a 10-fold gradient: 1 × 10^8^ copies/μL–1 copy/μL. NTC represents the negative control; Control represents the control line; Test represents the test line.

To optimize the assay’s performance, we assessed its analytical sensitivity by using different primer pairs and probe combinations. We used 10-fold serial dilutions of the template ranging from 1 copy/µL to 1 × 10^10^ copies/µL. Among the combinations tested, *env* primer + crRNA2 exhibited the highest sensitivity, with a detection limit of 10^1^ copies/µL (Figure 6). The *pol*2 primer + crRNA3 combination achieved a detection limit of 1 copy/µL (Figure 7) and was thus selected for the detection of field samples in subsequent experiments at a concentration of 1 copy/µL, which represented the method’s ultimate limit of detection (LOD).

### 3.4. Analytical Specificity of CRISPR-Cas13a-LF Assay

**Figure 8:**
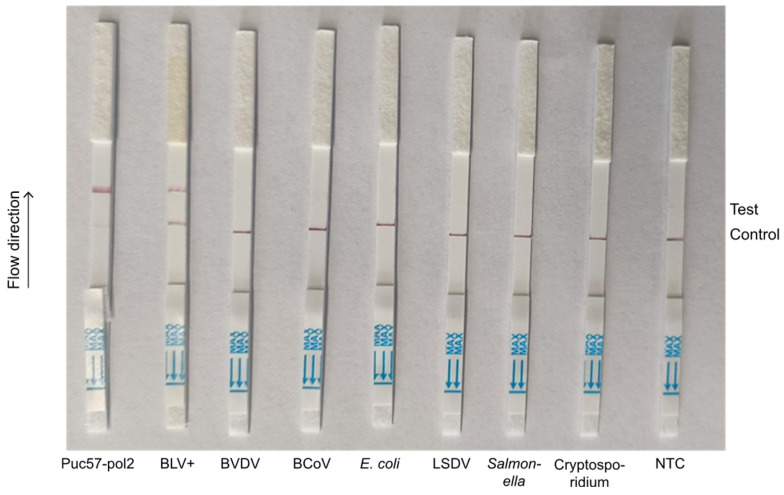
Specificity test of CRISPR-Cas13a-LF. pUC57-pol2 refers to *pol*2 plasmid. BLV^+^ indicates BLV-positive samples. The six common bovine pathogens considered were bovine viral diarrhea virus (BVDV), bovine coronavirus (BCoV), *Escherichia coli (E. coli)*, lumpy skin disease virus (LSDV), *Salmonella*, and *Cryptosporidium*. NTC represents the negative control. Control represents the control line. Test represents the test line.

To evaluate the specificity of the CRISPR-Cas13a-LF assay, we assessed its performance with six other bovine pathogens, BVDV, BCoV, *E. coli*, LSDV, *Salmonella*, and *Cryptosporidium*, by utilizing the *pol*2 + crRNA3 combination. The results demonstrate that the LF strips displayed negative bands in all cases, except for BLV, where we obtained a positive band (Figure 8). These findings highlight the high specificity of the method for detecting BLV.

### 3.5. Evaluation of CRISPR-Cas13a-LF on Field Blood Samples

**Figure 9:**
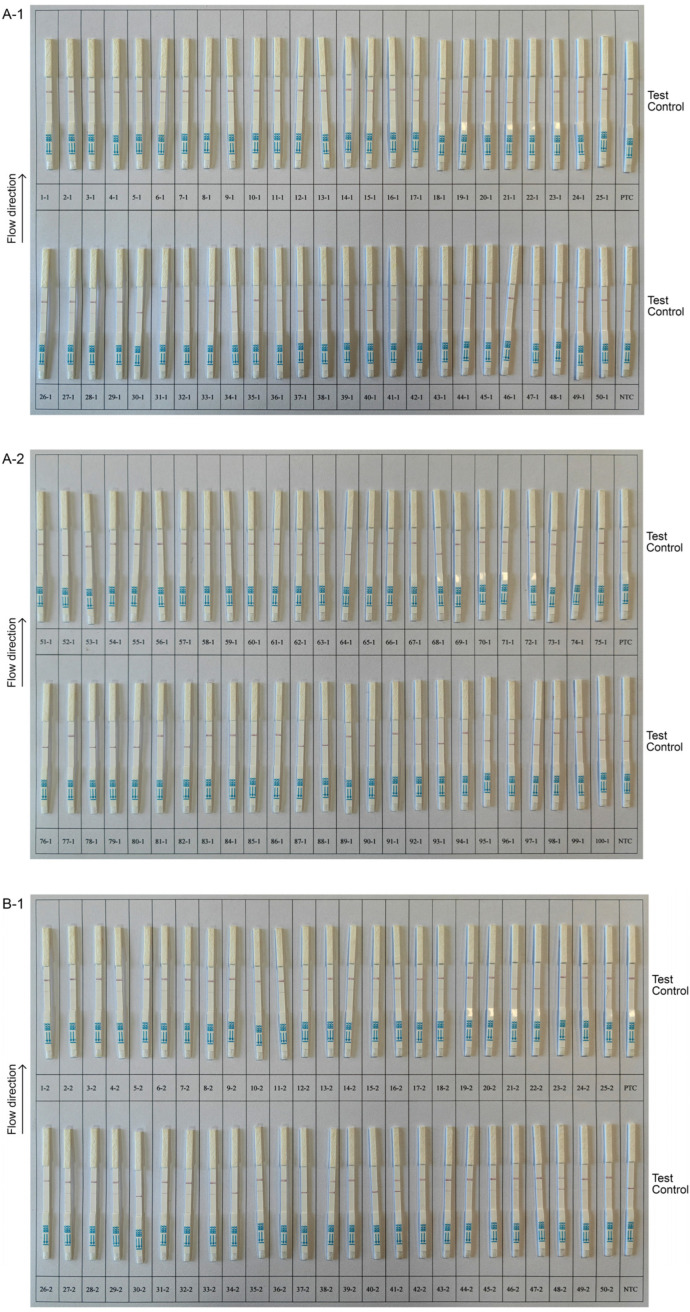

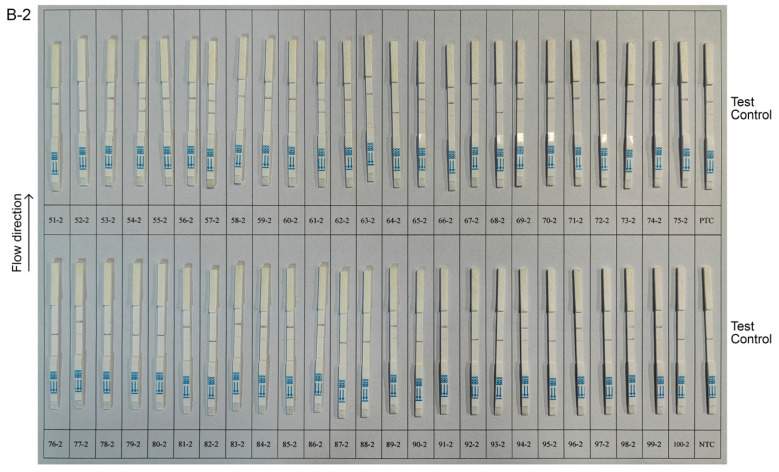
Clinical sample test with CRISPR-Cas13a-LF method. (**A-1**,**A-2**) First round of testing; (**B-1**,**B-2**) second round of testing. PTC represents positive control; NTC represents negative control.

To evaluate the efficacy of the CRISPR-Cas13a-LF assay on field blood specimens, a series of experiments were conducted. A total of 100 blood samples were tested in parallel with both BLV-CoCoMo-qPCR-2 and the CRISPR-Cas13a-LF method, which identified 78 positive samples and 22 negative samples. Our assay yielded identical outcomes to the baseline method, demonstrating 100% agreement (Table 3), indicating that our method has excellent differentiation between positive and negative samples. The Ct values for the BLV-CoCoMo-qPCR-2 method are shown in Supplementary File S2, while the results of the CRISPR-Cas13a-LF assay are shown in Figure 9.

To assess the reproducibility of the method, an independent replication of BLV detection was conducted by using all 100 field blood samples. The outcomes of both tests exhibited a high level of agreement, providing compelling evidence of the reliability and repeatability of the proposed methodology (Figure 9A-1,A-2,B-1,B-2). Hence, the CRISPR-Cas13a-LF method can serve as a dependable tool for BLV detection, obviating the need for costly equipment.

## 4. Discussion

The prevalence and impact of BLV in China present a complex epidemiological narrative, reflecting the country’s agricultural modernization and global integration. A systematic review and meta-analysis of studies from 1983 to 2019 estimated the overall BLV prevalence in China at 13.4% (4701/34,954) [33]. However, this figure masks significant temporal variations and disparities among cattle populations, with a recent study showing 49.1% prevalence in dairy cattle across 6 provinces and 1.6% in beef cattle across 15 provinces [34]. The temporal trajectory of BLV in China is characterized by initial detection and spread in the 1980s–1990s, followed by an apparent control period in the early 2000s and a subsequent resurgence from the mid-2000s onwards [33]. This resurgence is largely attributed to the intensification of international trade, particularly the importation of live cattle from BLV-prevalent countries. The virus has exerted substantial economic impacts on China’s burgeoning dairy industry, manifesting in reduced milk production and elevated culling rates [34]. As China becomes increasingly integrated into global agricultural markets, BLV control has gained importance for international trade in bovine and dairy products. This situation underscores the complex interplay between disease control efforts, agricultural practices, and international trade dynamics, highlighting the challenges of managing transboundary animal diseases in an era of globalized agriculture.

The economic implications of BLV infection in dairy herds are significant. Producers face an estimated average cost of USD 412 per case of lymphosarcoma, and the mean annual cost of subclinical infection, assuming 50% prevalence, reaches USD 6406 [35]. Importantly, eradicating BLV infection has never been deemed economically viable, particularly in the presence of high disease prevalence [36]. In China, the epidemiological situation regarding BLV infection is worrying, with the virus becoming widespread across the country [37]. Therefore, the implementation of measures to monitor and isolate animals infected with BLV is of utmost importance to preventing transmission among individuals.

Accurate and prompt disease diagnoses are crucial to preventing the spread of BLV. The BLV-CoCoMo-qPCR-2 assay is currently favored for its high sensitivity, specificity, and capability to detect and quantify diverse BLV strains globally However, despite its advantages, the high costs of qPCR instruments and the need for specialized personnel make it less suitable for routine clinical testing. Ideally, diagnostic methods should be affordable, accurate, efferent, and not require specialized equipment or personnel. Therefore, developing an accurate, efficient BLV detection method with low device dependence is crucial for early intervention and disease containment.

RAA-LF is a novel approach that combines the isothermal amplification of the target gene with the use of a labeled RAA probe, enabling the visual observation and determination of the amplified products with LF strips. This technology offers the advantage of not requiring expensive equipment and allows for the rapid and efficient amplification of the target fragment at a constant temperature. Significantly, the results of the amplified specimen can be directly visualized with the naked eye without the need for additional devices. However, it should be noted that the sensitivity of single RAA was found to be inadequate for the detection of low levels of target DNA [38]. Novel nucleic acid detection technologies employing CRISPR/Cas12 or Cas13 systems, coupled with RAA, have emerged as highly sensitive and specific methods [39].

In this study, we utilized RAA to effectively amplify the target DNA, which was then transcribed into RNA by employing the T7 RNA polymerase promoter, thereby facilitating the detection of LwCas13a. The ensuing fluorescence signal was meticulously tracked by means of LF visualization, thereby offering the convenience of direct observation with the unaided eye.

The efficacy of RAA-CRISPR-Cas13a in achieving specificity relies on the presence of mismatches within the crRNA target nucleotide region [40]. To assess this, we compared 20 different sequences of BLV. Based on the alignment results, we designed three sets of RAA primers and their corresponding crRNA, targeting the conserved nucleotide region of the *env*/*pol* gene. Among the three primer–probe combinations, we chose the most sensitive one for subsequent optimization.

The LOD achieved by the CRISPR-Cas13a-LF system was 1 copy/μL, highlighting its high sensitivity for detecting BLV. This method is more sensitive than BLV-LAMP, whose previously reported LOD was 2 copies/μL [41]. Simultaneously, the assay exhibited no cross-reactivity with six distinct bovine pathogens, thereby affirming its exceptional specificity—a crucial attribute in clinical testing.

To evaluate the clinical diagnostic reliability of CRISPR-Cas13a-LF for BLV, 100 field blood samples were analyzed, calculating the concordance between CRISPR-Cas13a-LF and BLV-CoCoMo-qPCR-2. Remarkably, the results revealed a 100% agreement between the two methods, indicating that CRISPR-Cas13a-LF exhibits high accuracy. Additionally, consistent outcomes from the two repeat tests demonstrate good reproducibility. These findings highlight the potential of CRISPR-Cas13a-LF as an efficient and accurate diagnostic tool for clinical cases of BLV.

In this study, the reaction time of the CRISPR-Cas13a-LF system was found to be 100 min. Following the extraction of DNA from bovine blood samples, the amplified *pol* gene underwent RAA and was subsequently transcribed into ssRNA within a 40 min incubation period at 37 °C. This process effectively activated the LwaCas13a nuclease. Within the same 40 min timeframe at 37 °C, the nucleases recognized the crRNA and cleaved the reporter gene. Finally, within a rapid 3–5 min timeframe, the appearance of a distinctive band on the strip indicated the presence of the reporter molecule. This test is well suited for implementation in locations where advanced equipment is unavailable.

This study has inherent limitations. Firstly, we failed to consider the crucial factor of nucleic acid extraction required for sample preparation, a pivotal step in on-site testing procedures. Secondly, while the CRISPR-Cas13a-LF assay exhibits notable advantages in terms of performance and accuracy, the costs associated with its fabrication, particularly the proprietary enzyme, pose significant limitations for large-scale pathogen detection, creating a trade barrier and impacting accessibility and affordability. To address this issue, potential solutions include optimizing enzyme production methods, identifying cost-effective alternatives, exploring open-source enzyme technologies, and integrating nucleic acid extraction into a more streamlined and automated platform. These strategies could reduce material costs and enhance the feasibility of large-scale testing in the future.

Our current CRISPR-13a detection method, while effective, involves multiple discrete steps that are procedurally complex. To overcome these challenges, we propose the development of an integrated, all-in-one detection platform that combines RAA, CRISPR technology, and LF tests. This innovative platform aims to streamline the entire detection process by consolidating sample preparation, amplification, CRISPR-based detection, and result readout into a single, seamless workflow. By leveraging a lateral flow strip format, we aim to create a rapid, sensitive, and user-friendly diagnostic tool tailored for point-of-care testing. Such integration could significantly reduce the detection time, minimize contamination risks, and simplify procedures for end-users, thus making advanced molecular diagnostics more accessible, especially in resource-limited settings. Future research will focus on addressing the technical challenges of merging these technologies, optimizing system performance, and validating its effectiveness across various pathogens and biomarkers. Ultimately, our goal is to enable faster, more accurate disease detection without the need for complex laboratory infrastructure or specialized technical expertise.

## 5. Conclusions

In conclusion, in this study, we effectively demonstrated the efficacy of CRISPR-Cas13a-LF as a sensitive, specific method for diagnosing BLV. The utilization of this assay shows significant potential in the field of BLV detection, offering an efficient approach for detecting and controlling the virus.

## Figures and Tables

**Table 1:** Information on primer molecular sequences.

Primer Names		Sequences (5′-3′)
Primer *pol* 1	BLV-pol1-F1	ACCTTCCCATGACTCAGGC
	BLV-pol1-R1	CTCCCGAGGCTTCGACTA
Primer *env*	BLV-env-F1	GGGGGCTTGATTGGTTGTACAT
	BLV-env-R1	GAACAGGCTTAGAACAGAA
Primer *pol* 2	BLV-pol2-F1	TGTCTCGATGGCCGAACCCACGTA
	BLV-pol2-R1	GGCATGAGTAGCTCCAGAGTAAG

**Table 2:** Information on primer and reporter molecular sequences.

Names	Sequences (5′-3′)
BLV-crRNA1	TTGCTGTCATTTCAGAGGGCGGAGAAAC
BLV-crRNA2	TGCGAGAGAGGCTGGAGATCACCGAGGC
BLV-crRNA3	GGGGCCCACCCTCTCTGAATAGTTCGCAT
RNA reporter	/56-FAM/mArArUrGrGrCmAmArArUrGrGrCmA/3 Bio/

**Table 3:** Comparison of BLV-CoCoMo-qPCR-2 and CRISPR-Cas13a-LF results from field samples.

Assay	Number of Samples	
	Positive	Negative
BLV-CoCoMo-qPCR-2	78	22
CRISPR-Cas13a-LF	78	22

## Data Availability

All data generated and analyzed during this study are incorporated within the manuscript.

## Supplementary Materials

The following supporting information can be downloaded at: https://www.mdpi.com/article/10.3390/ani14223262/s1, Supplementary File S1: pUC-*pol*1, pUC-*env*, and pUC-*pol*2 plasmid target gene sequence; Supplementary File S2: Ct values for 100 whole blood samples detected using the BLV-CoCoMo-qPCR-2 method.
