# Supplementary material for: CRISPR-Cas13a-Based Lateral Flow Assay for Detection of Bovine Leukemia Virus
Source: Animals (Basel). 2024 Nov 13;14(22):3262. doi: 10.3390/ani14223262 (PMC11590953; doi:10.3390/ani14223262)
Supplement: Supplementary file 1 [file animals-14-03262-s001.zip › Supplementary File S1.pdf]

*Pol* 1, *env pol* 2 plasmid gene sequence

> *pol*1 (GeneBank accession numbers: MH170028)
ACCTTCCCATGACTCAGGCCCTTTCTCGAGCCCTCTGGACTCACAATCAGA
TTAACCTCCTACCAATTCTAAAGACCAGATGGGAGCTACACCATTCACCCC
CACTTGCTGTCATTTCAGAGGGCGGAGAAACACCCAAGGGCTCTGATAAA
CTCTTTTTGTACAAGCTCCCCGGGCAAAACAATCGTCGGTGGCTAGGACCA
CTCCCGGCCCTAGTCGAAGCCTCGGGAG

> *env* (GeneBank accession numbers: LC733340)
GGGGGCTTGATTGGTTGTACATCCGGCTGGGTTTTCAAAGCCTATGTCCCA
CAATTAATGAGCCTTGCTGTTTCCTGCGCATTCAAAATGACTCCATTATCCG
CCTCGGTGATCTCCAGCCTCTCTCGCAAAGAGTCTCTACAGACTGGCAGTG
GCCCTGGAATTGGGATCTGGGGCTCACTGCCTGGGTGCGAGAAACCATTCA
TTCTGTTCTAAGCCTGTTC

> *pol*2 (GeneBank accession numbers: LC080651)
TGTCTCGATGGCCGAACCCACGTATCTCTGCCTGGGACCCCCGTTCCCCCG
CTACGCTGTGTGAAACCTGTCAAAAGCTTAATCCAACTGGAGGAGGAAAG
ATGCGAACTATTCAGAGAGGGTGGGCCCCGAATCATATTTGGCAGGCCGAT
ATAACCCATTATAAATACAAACAGTTCACCTACGCTCTGCATGTGTTTGTAG
ATACTTACTCTGGAGCTACTCATGCC
