# Supplementary material for: CRISPR-Cas13a-Based Lateral Flow Assay for Detection of Bovine Leukemia Virus
Source: Animals (Basel). 2024 Nov 13;14(22):3262. doi: 10.3390/ani14223262 (PMC11590953; doi:10.3390/ani14223262)
Supplement: Supplementary file 1 [file animals-14-03262-s001.zip › Supplementary File S2.pdf]

Table s1 Ct values for 100 whole blood samples detected using the BLV-CoCoMo-qPCR-2 method

Sample ID	Ct value	Sample ID	Ct value
1	25.23	51	24.61
2	26.31	52	40
3	21.31	53	22.84
4	23.02	54	23.97
5	23.84	55	24.2
6	22.72	56	23.22
7	24.79	57	24.39
8	23.99	58	22.55
9	22.13	59	25.46
10	25.02	60	24.25
11	25.74	61	25.41
12	25.18	62	25.97
13	23.04	63	23.26
14	23.14	64	40
15	24.18	65	25.1
16	21.61	66	26.3
17	23.67	67	25.9
18	25.59	68	40
19	22.59	69	24.38
20	22.5	70	23.96
21	40	71	25.4
22	40	72	24.45
23	23.45	73	25.12
24	24.23	74	24.69
25	25.54	75	25.22
26	23.35	76	40
27	22.22	77	25.1
28	22.01	78	40
29	22.17	79	40
30	40	80	40
31	24.75	81	25.15
32	24.44	82	40
33	23.07	83	25.4
34	40	84	25.17
35	25.02	85	25.84
36	25.25	86	25.98
37	40	87	40
38	24.02	88	40
39	24.29	89	23.82

40	40	90	40
41	25.4	91	26.06
42	23.68	92	40
43	26.24	93	40
44	22.7	94	23.84
45	22.99	95	40
46	22.65	96	26.37
47	22.3	97	40
48	22.52	98	26.03
49	22.9	99	23.83
50	22.18	100	40
PTC	23.5	PTC	23.5
NTC	40	NTC	40

Note: PTC: Positive Control; NTC: Negative Control; Ct: Cycle threshold value.
